# Florfenicol administration in piglets co-selects for multiple antimicrobial resistance genes

**DOI:** 10.1128/msystems.01250-24

**Published:** 2024-11-25

**Authors:** Devin B. Holman, Katherine E. Gzyl, Arun Kommadath

**Affiliations:** 1 Lacombe Research and Development Centre, Agriculture and Agri-Food Canada, Lacombe, Alberta, Canada; California State University Stanislaus, Turlock, California, USA

**Keywords:** microbiome, antimicrobial resistance, pigs, florfenicol, co-selection

## Abstract

Antimicrobial use in food-producing animals such as pigs is a significant issue due to its association with antimicrobial resistance. Florfenicol is a broad-spectrum phenicol antibiotic used in swine for various indications; however, its effect on the swine microbiome and resistome is largely unknown. This study investigated these effects in piglets treated intramuscularly with florfenicol at 1 and 7 days of age. Fecal samples were collected from treated (*n* = 30) and untreated (*n* = 30) pigs at nine different time points up until 140 days of age, and the fecal metagenomes were sequenced. The fecal microbiomes of the two groups of piglets were most dissimilar in the immediate period following florfenicol administration. These differences were driven in part by an increase in the relative abundance of *Clostridium scindens*, *Enterococcus faecalis*, and *Escherichia* spp. in the florfenicol-treated piglets and *Fusobacterium* spp., *Pauljensenia hyovaginalis*, and *Ruminococcus gnavus* in the control piglets. In addition to selecting for florfenicol resistance genes (*floR*, *fexA*, and *fexB*), florfenicol also selected for genes conferring resistance to the aminoglycosides, beta-lactams, or sulfonamides up until weaning at 21 days of age. Florfenicol-resistant *Escherichia coli* isolated from these piglets were found to carry a plasmid with *floR*, along with *tet*(A), *aph(6)-Id*, *aph(3″)-Ib*, *sul2*, and *bla*_TEM-1_/*bla*_CMY-2_. A plasmid carrying *fexB* and *poxtA* (phenicols and oxazolidinones) was identified in florfenicol-resistant *Enterococcus avium*, *Enterococcus faecium*, and *E. faecalis* isolates from the treated piglets. This study highlights the potential for co-selection and perturbation of the fecal microbial community in pre-weaned piglets administered florfenicol.

**IMPORTANCE:**

Antimicrobial use remains a serious challenge in food-animal production due to its linkage with antimicrobial resistance. Antimicrobial resistance can reduce the efficacy of veterinary treatment and can potentially be transferred to humans through the food chain or direct contact with animals and their environment. In this study, early-life florfenicol treatment in piglets altered the composition of the fecal microbiome and selected for many unrelated antimicrobial resistance genes up until weaning at 21 days of age. Part of this co-selection process appeared to involve an *Escherichia coli* plasmid carrying a florfenicol resistance gene along with genes conferring resistance to at least four other antimicrobial classes. In addition, florfenicol selected for certain genes that provide resistance to multiple antimicrobial classes, including the oxazolidinones. These results highlight that florfenicol can co-select for multiple antimicrobial resistance genes, and their presence on mobile genetic elements suggests the potential for transfer to other bacteria.

## INTRODUCTION

In commercial swine production, antimicrobials are frequently used to both treat and prevent infectious disease. These antimicrobials are usually administered via feed, water, or intramuscular injection. However, antimicrobial use can be associated with the development and maintenance of antimicrobial resistance in pigs and may also affect the gut microbiome. Antimicrobial resistance is a serious threat to both animal and human health as it can reduce the efficacy of veterinary antimicrobials, and antimicrobial-resistant bacteria may be transferred to humans through food or direct contact with animals and their environment (1). Florfenicol is a broad-spectrum phenicol antibiotic that inhibits bacterial protein synthesis by binding to the 50S ribosomal subunit (2). Although florfenicol is only used in food-producing animals, it is structurally similar to chloramphenicol, which is used in human medicine. Unlike some older antimicrobials such as chlortetracycline and tylosin, florfenicol is a relatively recent addition to veterinary medicine and was only approved for use in swine in Canada in 2004.

In pigs, florfenicol is typically delivered via intramuscular injection and is indicated for the treatment of respiratory disease associated with bacteria such as *Actinobacillus pleuropneumoniae*, *Bordetella bronchiseptica*, *Glaesserella parasuis*, *Pasteurella multocida*, and *Streptococcus suis* and may also be used off-label for other purposes (3). Intramuscular injection of florfenicol (15 mg/kg body weight) in pigs, with a plasma half-life of 11–18 h (4, 5), has been shown to result in concentrations of the drug in the lower gastrointestinal (GI) tract that are similar to or higher than that of orally dosed pigs (6). Resistance to florfenicol is often mediated through phenicol exporter genes such as *fexA*, *fexB*, or *floR* (2, 7) as well as the ribosomal protection protein genes *optrA* and *poxtA* and the 23S rRNA methyltransferase gene *cfr* (chloramphenicol-florfenicol resistance) (8). The *optrA* and *poxtA* genes are of particular concern since they also provide resistance to the oxazolidinones, which are classified as critically important in human medicine (9). Significantly, many of the genes conferring resistance to florfenicol are carried on mobile genetic elements (MGEs) such as plasmids and transposons, which enhance their dissemination among bacteria (10).

The gut microbiome plays an important role in the health and development of pigs, providing energy in the form of short-chain fatty acids from otherwise non-digestible carbohydrates (11), aiding in the maturation of the immune system (12), and resisting colonization by pathogens (13), among other benefits. Microbial colonization of the piglet GI tract begins at birth as they are exposed to microbes from the sow (colostrum/milk, feces, and vaginal tract) and their surrounding environment. Initially, the pig gut is dominated by bacterial genera such as *Clostridium*, *Enterococcus*, and *Escherichia* during the early nursing period (14), with a rapid transition to *Bacteroides* and *Lactobacillus* spp. followed by an increase in the abundance of *Faecalibacterium*, *Megasphaera*, and *Prevotella* spp. post-weaning (15). The gut microbiome is then influenced by factors such as diet, age, and antimicrobial exposure (15, 16). Because the nursing piglet gut microbiome is less stable than that of older pigs (15, 17), antimicrobial interventions may have a larger effect on the microbiome during this phase.

To date, there are no published studies that have investigated the effect of florfenicol on the fecal microbiome or resistome (all antimicrobial resistance genes [ARGs]) in pigs. At our swine facility, florfenicol is routinely given to male piglets after castration to prevent infection. Therefore, in this study, we administered florfenicol to piglets and collected fecal samples at several different time points until they reached market weight to determine the short- and long-term effects of florfenicol on the fecal microbiome and resistome. We also collected colostrum, milk, vaginal, and fecal samples from their sows to assess the contribution of these sources to the piglet microbiome and resistome.

## MATERIALS AND METHODS

### Animals and experimental design

Pigs were cared for according to the guidelines of the Canadian Council on Animal Care. Landrace × Yorkshire sows (*n* = 14) that had been inseminated with Duroc semen were given oxytocin to ensure that they all farrowed around the same time. None of the sows received any antimicrobials during the gestation or nursing period. The sows had been vaccinated against *E. coli* (strains K99, K88, 987P, and F41) and *Clostridium perfringens* Type C (LitterGuard LT-C; Zoetis Inc., Kalamazoo, MI, USA), as well as porcine parvovirus, *Erysipelothrix rhusiopathiae*, *Leptospira bratislava*, *Leptospira canicola*, *Leptospira grippotyphosa*, *Leptospira hardjo*, *Leptospira icterohaemorrhagiae*, and *Leptospira pomona* (Farrowsure Gold, Zoetis Inc.).

Each litter was randomly assigned to either the florfenicol treatment (*n* = 7) or the control (*n* = 7) group. Within each litter, two piglets per sex were randomly assigned to the florfenicol (*n* = 30) or the control (*n* = 30) group. At 1 and 7 days of age, piglets in the florfenicol treatment group were given an intramuscular injection of florfenicol (15 mg/kg body weight). Pigs from the two treatment groups had no contact with each other during the study and piglets consumed only sow milk prior to weaning at 21 days of age (i.e., no creep feed). At weaning, piglets within each treatment group were randomly assigned to a pen with four other piglets. The individual pig was considered the experimental unit. Post-weaning, all pigs received the same diet that was free of antimicrobials (Table S1).

Fecal swabs (FLOQSwabs; Copan, Murrieta, CA, USA) were collected from piglets at 1, 4, 7, 11, 14, 21, 28, 84, 112, and 140 days of age. To assess the contribution of the sow to the piglet fecal microbiome and resistome, vaginal swabs were taken from the sows at farrowing (day 0) and fecal swabs on days 0, 1, 4, 7, 11, 14, and 21. Colostrum was collected from the sows during or immediately after farrowing, and milk was collected on days 7 and 21. Approximately 30 min prior to the collection of milk, sows were injected with oxytocin to induce milk expression. The teat was thoroughly cleaned with 0.5% hydrogen peroxide prior to the collection of colostrum and milk into a sterile 150-mL screw cap container using sterile gloves. All samples were immediately placed on ice, transported to the laboratory, and stored at −80°C until DNA extraction. Pigs were also weighed on days 1, 7, 14, 21, 28, 70, 84, 112, and 140.

### Extraction of DNA

DNA was extracted from fecal and vaginal swabs using the QIAamp BiOstic bacteremia DNA kit (Qiagen, Mississauga, ON, Canada) as previously described (15). The DNeasy PowerFood Microbial kit (Qiagen) was used to extract DNA from the colostrum and milk samples. Fat was removed from the sample with a sterile cotton swab after centrifugation at 5,000 × *g* for 30 min at 4°C. The pellet was washed with 1.8 mL of phosphate-buffered saline and centrifuged at 10,000 × *g*, and the remaining fat layer was removed. The wash step was repeated three to five times until all the visible fat was removed. The pellet was subjected to enzymatic lysis with 90 µL of 50-mg/mL lysozyme (Roche, Mannheim, Germany) and 50 µL of 5-kU/mL mutanolysin from *Streptomyces globisporus* American Type Culture Collection (ATCC) 21553 (MilliporeSigma, Oakville, ON, Canada) at 55°C for 15 min followed by the addition of 28 µL of 20-mg/mL proteinase K (MilliporeSigma) for an additional 15 min at 55°C. The suspension was then centrifuged at 10,000 × *g* for 1 min at room temperature, and the supernatant was discarded. The pellet was suspended in 450-µL MBL buffer (Qiagen) and transferred to a PowerBead tube (Qiagen). The PowerBead tube was vortexed for 5 s and then placed into a thermoshaker set at 65°C and 1,400 rpm for 10 min. Following incubation, the samples were bead-beaten in an MP FastPrep-24 (MP Biomedicals, Solon, OH, USA) at 6 m/s for 90 s. After a 5-min rest, the samples were centrifuged at 13,000 × *g* for 1 min at room temperature. The supernatant was then transferred to a clean collection tube, and the manufacturer’s protocol was followed for all subsequent steps.

### 16S rRNA gene sequencing and analysis

The V4 region of the 16S rRNA gene in the extracted DNA from all fecal, colostrum/milk, and vaginal samples was amplified and sequenced as previously described (18). Cutadapt v.3.4 (19) was used to remove primer sequences and any reads shorter than 215 bp. The reads were then processed using DADA2 v.1.20.0 (20) in R v.4.1.0 with forward and reverse reads trimmed to 200 bp each and merged with a minimum overlap of 75 bp. Amplicon sequence variants (ASVs) were resolved; chimeras were removed; and taxonomy was assigned to the ASVs using the SILVA SSU database release 138.1 (21). ASVs classified as chloroplasts, mitochondria, or eukaryota were removed. Extraction kit controls (*n* = 18) were also included. However, only one ASV classified as *Escherichia coli* was identified in these negative controls and at a significantly lower abundance (30× lower) than in the biological samples and was therefore retained. A 20-strain whole-cell mock community was also extracted and sequenced (MSA-2002, ATCC) as a positive control. The pig fecal samples were rarefied to 5,200 sequences prior to the calculation of ASV richness, and the inverse Simpson diversity as well as Bray-Curtis dissimilarities using Phyloseq v.1.36.0 (22) and vegan 2.6-4 (23). The average number of sequences per sample was 12,341 ± 155 SEM, and a rarefaction level of 5,200 sequences was used to maximize the number of samples included in the analyses (9 samples lost out of 572).

### Shotgun metagenomic sequencing and analysis

Fecal metagenomic DNA from a random subset of 16 pigs within each treatment group (one pig per sex per litter) from days 1, 4, 7, 11, 14, 21, 28, 84, and 140 were selected for shotgun metagenomic sequencing to provide higher resolution taxonomy and functional information. All sow fecal samples from days 1, 7, and 21 as well as all colostrum/milk and vaginal swabs collected were also included. Metagenomic libraries were prepared and sequenced on a NovaSeq 6000 instrument with a S4 flow cell (300 cycles) (Illumina Inc., San Diego, CA, USA) as previously detailed (24). Low-quality reads and sequencing adapters were removed with fastp v.0.23.2 (25) using a 4-bp sliding window and a quality threshold of 15. Reads shorter than 100 bp were also removed. To remove swine host and PhiX sequences, the reads were aligned to three swine genome assemblies (Sscrofa11.1 [Duroc], Berkshire_v1 [Berkshire], and USMARC v.1.0 [Duroc × Landrace × Yorkshire]) and the *Escherichia* phage phiX174 genome (NC_001422) with Bowtie2 v.2.4.4 (26). Reads that did not map to these genomes were then extracted using SAMtools v.1.14 (27) and BEDtools v.2.30.0 (28). The metagenomic reads were taxonomically classified using Kraken2 v.2.1.3 (29) and Bracken v.2.9 (30) with the Genome Taxonomy Database (GTDB) release 220 (31). The Resistance Gene Identifier (RGI) v.6.0.2 and the Comprehensive Antibiotic Resistance Database (CARD) v.3.2.6 (32) with KMA (33) were used to screen the unassembled reads for ARGs.

### Metagenome-assembled genomes

The metagenomic reads were individually assembled for each sample as well as co-assembled, depending on the sample type and treatment group using MEGAHIT v.1.2.9 (34). Briefly, for the co-assembly step, only similar samples were assembled together. Specifically, these groups of samples were the colostrum/milk, vaginal, sow fecal, pre-weaning control, pre-weaning florfenicol, post-weaning control, and post-weaning florfenicol fecal samples. Each sample was mapped back to its respective assembly and co-assembly using Bowtie2, and the contigs (≥2,000 bp) were binned into metagenome-assembled genomes (MAGs) using MetaBAT 2 v.2.2.15 (35). The completeness and contamination of the co-assembled and individually assembled MAGs were calculated with CheckM v.1.2.0 (36) resulting in 78,968 MAGs that were at least 90% complete and had less than 5% contamination. These MAGs were then dereplicated using dRep v.3.2.2 with primary clustering set at 90% average nucleotide identity (ANI) and secondary clustering set at 99% ANI. Taxonomy was then assigned to the 1,546 dereplicated MAGs using GTDB-Tk v.2.4.0 (37) and GTDB release 220.

Antimicrobial resistance genes were identified in the MAGs using the RGI with the CARD. *E. coli* MAGs were classified by serotype using SerotypeFinder v.2.0 (38), and multilocus sequence typing was done on MAGs assigned to a species that was in the PubMLST database (39). PhyloPhlAn v.3.0.67 (40) was used to create a phylogenomic tree of the MAGs by aligning 400 universal marker genes. The relative abundance of each MAG in each sample was calculated using CoverM v.0.6.1 (https://github.com/wwood/CoverM).

### Quantification of *floR*

As *floR* was the most differentially abundant ARG between the treated and untreated pigs, the absolute concentration of this gene was determined using quantitative PCR (qPCR). Briefly, the primers *floR*-F 5′-CGGTCGGTATTGTCTTCACG-3′ and *floR*-R 5′-TCACGGGCCACGCTGTAT-3′ (41) were used to target and amplify the *floR* gene in the same samples previously selected for shotgun metagenomic sequencing. Each reaction contained the forward (7.5 pmol) and reverse primers (5 pmol), 25 to 50 ng DNA template, and Brilliant II SYBR Green qPCR Master Mix (Agilent Technologies, Mississauga, ON, Canada) in a total volume of 20 µL. The three-step PCR consisted of an initial denaturation at 95°C for 10 min followed by 40 cycles of annealing at 54°C for 1 min and extension at 72°C for 30 s as per manufacturer’s instructions. Fluorescence was detected during annealing and extension on an AriaMx real-time PCR system (Agilent Technologies). A dissociation curve was generated at the end of each qPCR run with an increase in temperature of 0.5°C/s until it reached 95°C to ensure that only one amplicon was produced. A negative control and a serially diluted seven-point standard curve from 1.42 × 10^2^ to 1.42 × 10^8^ copies were included with each qPCR run. The standard consisted of the *floR* gene in a pUCIDT (Amp) vector (Integrated DNA Technologies, Coralville, IA, USA). The DNA concentration of the *floR* standard was determined with a dsDNA HS assay kit (Thermo Fisher Scientific, Waltham, MA, USA). Copy number was calculated using the stock *floR* standard concentration, the plasmid size, and a molar mass of 650 g/mol/bp. All samples and standards were analyzed in triplicate.

### Culturing and sequencing of florfenicol-resistant bacteria

Florfenicol-resistant isolates were recovered from fecal swabs (*n* = 11) from florfenicol-treated piglets on days 4 and 11 to evaluate the genomic context of certain florfenicol resistance genes. Briefly, swabs were added to 10 mL of brain heart infusion (BHI) broth (Oxoid, Basingstoke, Hampshire, UK) in Hungate anaerobic tubes (Chemglass Life Sciences, Vineland, NJ, USA) and incubated for 4 h at 39°C (pig body temperature). Serial dilutions were then plated onto BHI agar (Oxoid) containing 32-µg/mL florfenicol (Thermo Fisher Scientific) and incubated for 24 h at 39°C under anaerobic conditions. This is at least twice the concentration for published minimum inhibitory concentration (MIC) breakpoints for florfenicol resistance in animal isolates to ensure the recovery of florfenicol-resistant bacteria (42). One colony was selected from each agar plate, re-streaked onto BHI agar with 32-µg/mL florfenicol, and incubated at 39°C for 16 h. From these plates, one colony was inoculated into 10 mL of BHI broth in a Hungate tube and incubated for 6 h at 39°C. These cultures were then stored in BHI with 20% (vol/vol) glycerol at −80°C until DNA extraction.

DNA was extracted from the cultures; sequence libraries were prepared; and the genome libraries were sequenced on a MiSeq instrument with a MiSeq Reagent Kit v.2 (300 cycles, 2 × 150 bp) (Illumina Inc.) as previously described (43). The florfenicol-resistant isolates were also sequenced using a MinION Mk1B device with an R10.4.1 flow cell (Oxford Nanopore Technologies, Oxford, UK). Briefly, high-molecular-weight (HMW) DNA was extracted from an overnight culture using the Monarch HMW DNA Extraction Kit for Tissue (New England Biolabs, Ipswich, MA, USA) as per manufacturer’s protocol. The DNA concentration was determined with a Qubit dsDNA HS assay kit, and the size and quality of the DNA were evaluated with a genomic DNA ScreenTape system (Agilent Technologies) as per manufacturer’s instructions. Library preparation was done with the Native Barcoding Kit 24 v.14 following the protocol supplied by the manufacturer. HMW DNA from *E. coli* was difficult to elute from the AMPure XP Beads after the DNA repair and end-prep step; therefore, the input DNA concentration was increased from 400 ng to 1 µg, the volume of nuclease-free water to solubilize the HMW DNA was increased from 10 µL to 20 µL, and the incubation time was increased from 2 min to 10 min.

During the adapter ligation and cleanup step, the long fragment buffer supplied in the kit was used to enrich for DNA fragments over 3 kb in length. For each flow cell, the recommended amount of final prepared library was loaded together with the library beads supplied in the kit. The flow cell was primed with the recommended addition of ultrapure bovine serum albumin (Invitrogen, Waltham, MA, USA) to a final concentration of 0.2 mg/mL. During data acquisition and base calling, the default parameters were used in the MinKNOW v.23.11.4 software installed with Bream v.7.8.2, Dorado v.7.2.13, and MinKNOW core v.5.8.6. After the sequencing run was completed, base calling was re-done with the high accuracy model within MinKNOW.

### Genomic analysis of florfenicol-resistant isolates

Genomes were assembled using the MinION reads and polished with Illumina reads as per Wick et al. (44). Briefly, all MinION reads were filtered with Filtlong v.0.2.1 (https://github.com/rrwick/Filtlong), removing reads shorter than 6,000 bp and retaining only the best 90% of reads. Filtered reads for each sample were then subsampled into 12 read subsets with Trycycler v.0.5.4 (45), and an assembly was made with each read subset. The four assemblers used were Flye v.2.9.2-b1786 (46) with the nano-hq flag, miniasm v.0.3-r179 (47) with minimap2 v.2.26-r1175 (48), and polishing by minipolish v.0.1.2 (49), Raven v.1.8.3 (50) with Racon v.1.5.0 (51) and the disable-checkpoints flag, and Canu v.2.2 (52). All contigs generated from each of the assemblers for each sample were then grouped into clusters using Trycycler. Clusters were discarded if most contigs in the cluster did not have a similar size and depth. “Trycycler reconcile” was run on each cluster with contigs and clusters removed if they could not be reconciled.

A multiple sequence alignment was made from each reconciled cluster with “Trycycler msa,” and then each read was assigned to a cluster using “Trycycler partition.” A consensus sequence was then generated from each cluster with “Trycycler consensus.” The consensus sequence for each cluster was polished with Medaka v.1.11.1 (https://github.com/nanoporetech/medaka) using the r1041_e82_400bps_hac_v4.2.0 model. The polished assemblies were concatenated into one assembly per sample. Prior to polishing the long-read assemblies with Polypolish v.0.5.0 (53), the Illumina reads were filtered with fastp, aligned to the polished assembly for each sample with bwa v.0.7.17-11 (54), indexed with bwa, and size filtered with Polypolish. Taxonomy was assigned to the polished genome assemblies with the GTDB-Tk and GTDB release 220. Assembly metrics were assessed with QUAST v.5.2.0 (21), and contamination and completeness were determined with CheckM2 v.1.0.1 (55). The finished genomes were screened for ARGs with the CARD RGI, and the *E. coli* isolates were assigned to serotypes as described above for the MAGs. Plasmids from the polished genomes were classified based on their replicon type using PlasmidFinder v.2.1.6-1 (56). The ANI between genomes was calculated with fastANI v.1.33 (57), and the isolate genomes and their associated plasmids were viewed with Proksee (58).

The susceptibility of the *Enterococcus* isolates to linezolid was also assessed based on their ARG profiles and the importance of linezolid in human medicine. Briefly, linezolid (MilliporeSigma) was dissolved in molecular-grade water to a concentration of 2.5 mg/mL and then diluted in cation-adjusted Mueller-Hinton (MH) broth 2 (MilliporeSigma) to a concentration of 128 µg/mL. This linezolid working stock was serially diluted twofold into designated wells in a 96-well microplate (Thermo Fisher Scientific). To prepare the inoculum for each isolate, the culture was streaked onto BHI agar and grown overnight (16 h) at 39°C. Colonies from the plate were then added to MH broth until the suspension resembled a 0.5 McFarland standard, and 100 µL of this suspension was added to 9.9 mL of MH broth. Finally, 50 µL of this inoculum was added to the designated wells. The inoculated microplate was grown for 48 h at 35°C, and the lowest linezolid concentration without visible growth was determined to be the MIC. The susceptibility assay was performed in duplicate on 3 separate days. Two enterococci strains known not to be carrying any oxazolidinone resistance genes, *Enterococcus faecium* ATCC 35667 and *Enterococcus faecalis* ATCC 29212, were included as controls. Interpretation of the MICs was based on the Clinical and Laboratory Standards Institute (CLSI) breakpoints of ≤2 µg/mL = sensitive, 4 µg/mL = intermediate resistance, and ≥8 µg/mL = resistant (59).

### Statistical analysis

Differentially abundant microbial species (metagenomic data), ARGs, and MAGs between the florfenicol-treated and control pigs were identified with MaAsLin2 v.1.16.0 (60) in R v.4.3.2 using a general linear model. Only those microbial species with a percent relative abundance of at least 0.1% and ARGs present in at least 25% of the samples being analyzed were included. Permutational multivariate analysis of variance (PERMANOVA) of the Bray-Curtis dissimilarities was calculated in vegan to determine the effect of florfenicol treatment on the overall composition of the microbial composition (ASVs from 16S rRNA gene data) and resistome (ARGs). Non-metric multidimensional scaling was used to display the Bray-Curtis dissimilarities. The effect of florfenicol on average daily gain was evaluated using a linear mixed model with individual pig as the random effect and treatment and age as the fixed effects with the R package lme4 v.1.1-32 (61). Post hoc comparisons were carried out within each time period using Tukey’s honestly significant difference. Sourcetracker2 v.2.0.1-0 (62) was used to predict the relative contribution of the sow vagina, colostrum, milk, and feces to the piglet fecal microbiome. For this analysis, the samples included were limited to those from the control piglets and their sows to avoid any treatment effect. The 16S rRNA gene sequences, rarefied to 2,000 sequences per sample, were used to assess the contributions to the microbiome. This rarefaction level was chosen to retain as many samples as possible as the colostrum/milk samples in particular had fewer sequences than the other sample types (6,662 ± 363). The ARG data (relative abundance) from the unassembled reads were used for the resistome assessment. The feces of the piglets were considered the sink, and the sow colostrum, milk, vaginal, and feces were the sources included.

## RESULTS

### Animal performance

The average daily gain differed significantly between the control and florfenicol-treated pigs only during the 1- to 7-day period (Fig. S1). Notably, it was the florfenicol-treated piglets that grew slower during this time frame (0.20 ± 0.01 kg/day vs 0.26 ± 0.01 kg/day). As expected, the average daily gain for piglets was reduced during the 1-week post-weaning period as they adjusted to solid feed.

### Metagenomic sequencing summary

After quality filtering and host DNA removal, 1.85 Tb of sequence data from 368 metagenomic samples were available for downstream analyses. The number of metagenomic reads per sample did not differ between the florfenicol and control piglets (*P* > 0.05). As expected, given the difficulty in removing host cells from colostrum and milk samples prior to DNA extraction, these samples had a very low microbial-to-host DNA ratio (up to 99% host DNA), and vaginal swabs were also heavily contaminated with DNA from the sow (Table S2). The metagenomes of the mock community were also assembled and binned into MAGs. All 20 bacterial species in the mock community were represented among these MAGs, although with varying levels of completeness, and no species not included in the mock community were identified (Table S3).

### Effect of florfenicol on the piglet fecal microbiome

**Fig 1:**
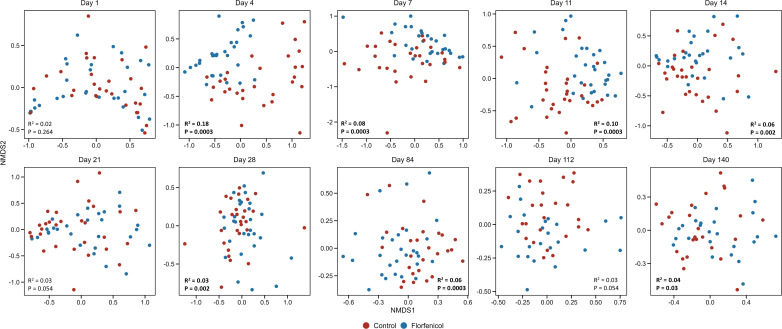
Non-metric multidimensional scaling (NMDS) plots of the Bray-Curtis dissimilarities for untreated pigs (control, *n* = 30) and pigs treated with florfenicol (*n* = 30) on days 1 and 7 based on 16S rRNA gene sequences. Permutational multivariate analysis of variance (PERMANOVA) *R*^2^ and *P* values are included in each plot.

Florfenicol administration had the greatest effect on the piglet fecal microbial community structure on day 4, which was 3 days after the first treatment, based on the 16S rRNA gene sequence data (PERMANOVA: *R*^2^ = 0.18, *P* = 0.0003; Fig. 1). A second injection on day 7 increased the dissimilarity of the microbiomes of the two groups of pigs; however, this effect was lost by day 21, just prior to weaning. Although the effect was relatively small, the control and florfenicol-treated pigs did have significantly different microbiomes on days 28, 84, and 140, suggesting there may have been some minor residual effects of florfenicol treatment post-weaning. The florfenicol-treated piglets had a less rich (fewer ASVs) microbiome on days 4, 11, and 28, although the opposite was true at the end of the study (day 140) (Fig. S2). In terms of microbial diversity, the control piglets had greater microbial diversity (inverse Simpson diversity index) than the florfenicol group only on day 28 (Fig. S2).

**Fig 2:**
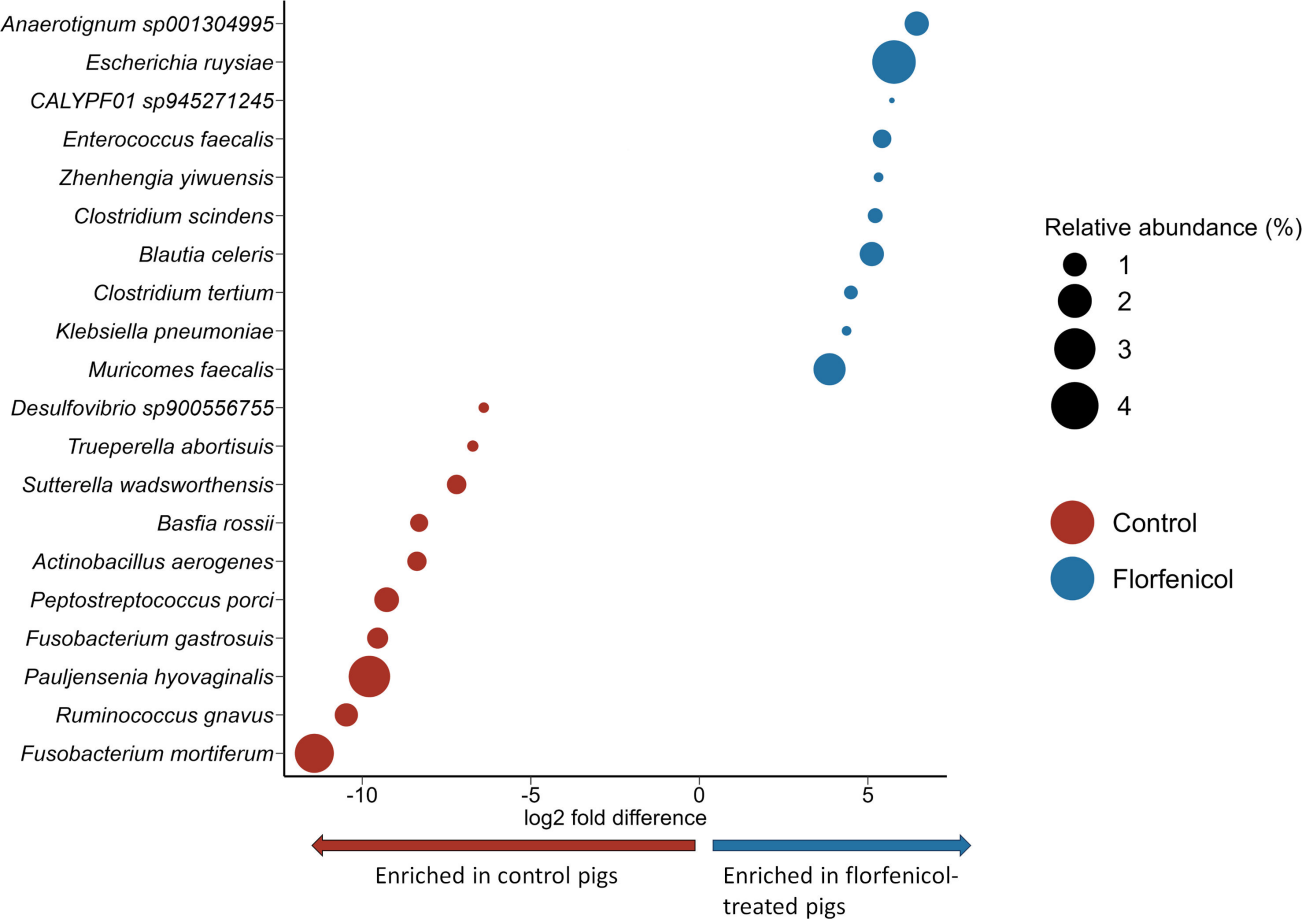
The 20 most differentially abundant bacterial species between control (*n* = 16) and florfenicol-treated (*n* = 16) piglets at 4 days of age. The size of the circles is proportional to the relative abundance (%).

**Fig 3:**
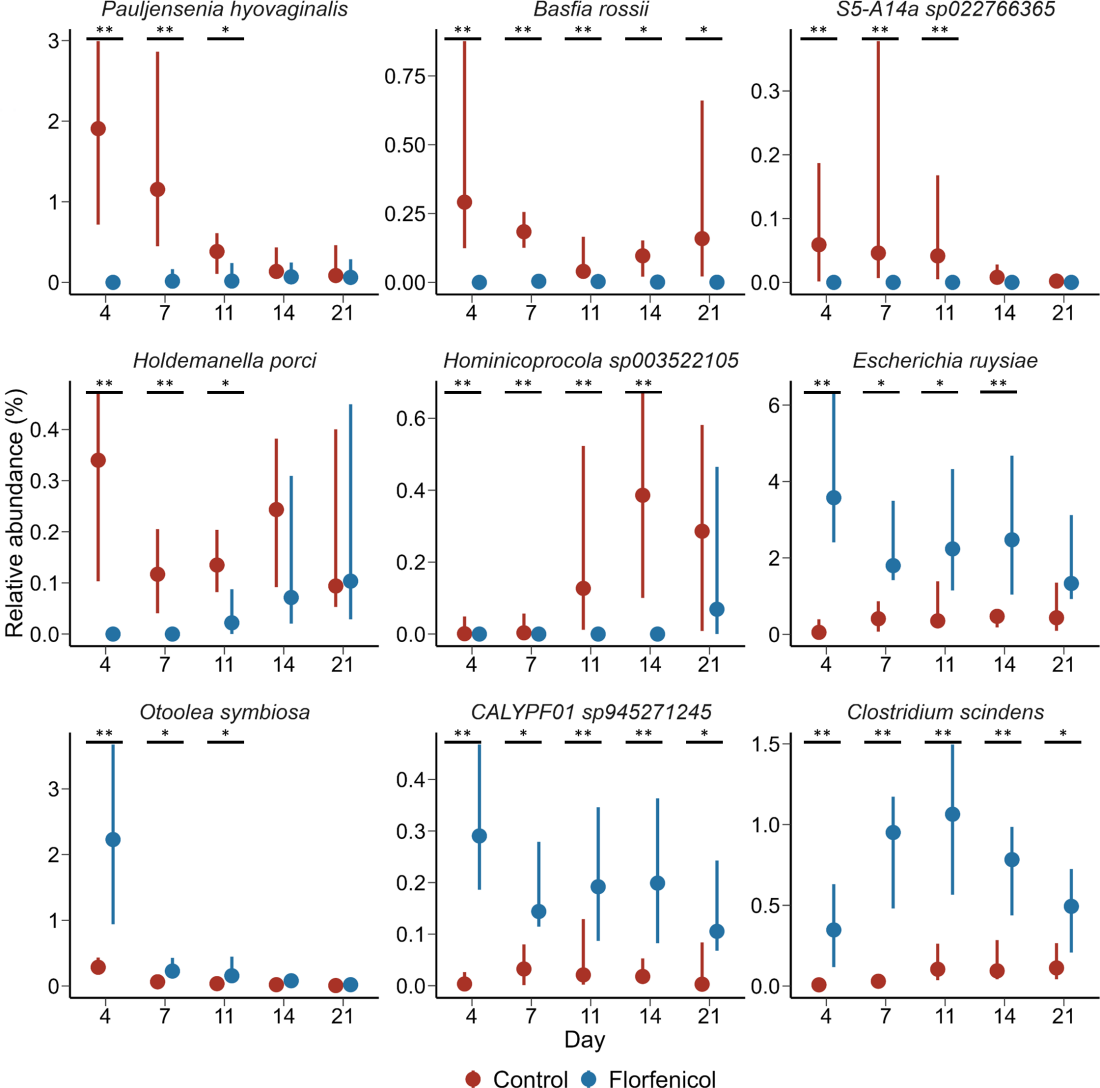
Percent relative abundance of bacterial species that were consistently differentially abundant between control (*n* = 16) and florfenicol-treated (*n* = 16) piglets on days 4, 7, and 11 (*n* = 16). The point represents the median value, and the lines are the 25th and 75th percentiles. *P < 0.05, **P <0.01.

Based on the analysis of the metagenomic sequences, there were 67 bacterial species that were differentially abundant between the control and florfenicol-treated pigs on day 4 (Fig. 2; Table S4). *Fusobacterium mortiferum*, *Ruminococcus gnavus*, and *Pauljensenia hyovaginalis* were among the species strongly associated with the control pigs at this time, while *Anaerotignum* sp001304995, *Escherichia ruysiae*, and *E. faecalis* were among the 28 bacterial species relatively more abundant in pigs treated with florfenicol. There were progressively fewer bacterial species that were differentially abundant between the two groups of pigs from days 7 through 21, just prior to weaning. Nine bacterial species were consistently differentially abundant between the control and florfenicol-treated pigs on days 4, 7, and 11 (Fig. 3). Among these species, *Basfia rossii* (National Center for Biotechnology Information [NCBI]: *Actinobacillus rossii*) also had a greater relative abundance in the fecal microbiomes of the control pigs from day 4 through 21, while CALYPF01 sp945271245 and *Clostridium scindens* were associated with the florfenicol-treated pigs during this period. Although no bacterial species were differentially abundant between the two groups at 1 week post-weaning (day 28) and at the end of the study (day 140), 12 species were differentially abundant on day 84 (Table S4). There was also one species, *Holdemanella porci*, that was significantly associated with the control pigs on day 84 in addition to days 4, 7, and 11.

### Effect of florfenicol on the piglet fecal resistome

**Fig 4:**
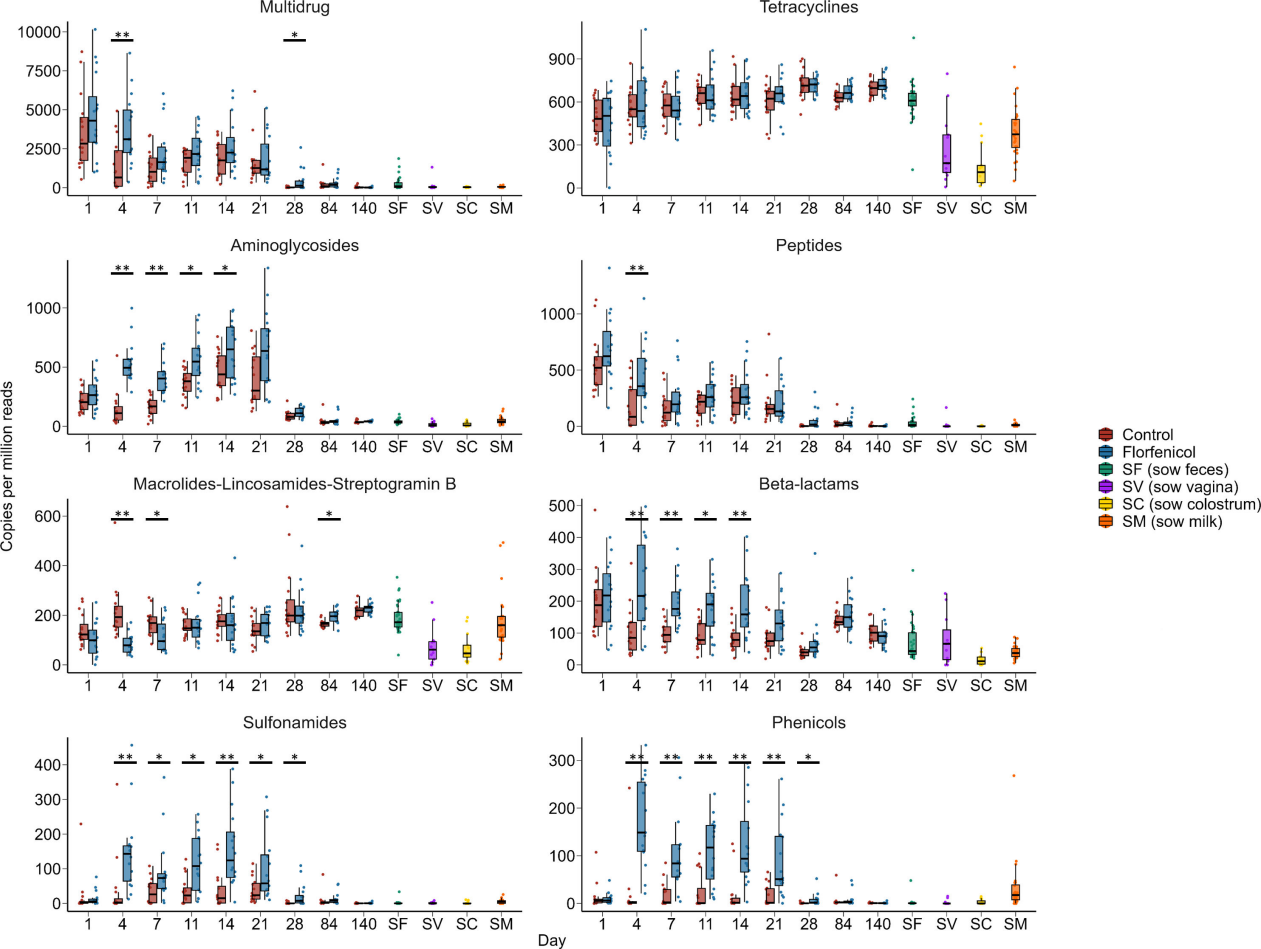
The relative abundance of antimicrobial resistance genes by antimicrobial class they confer resistance in the metagenomes of control (*n* = 16) and florfenicol-treated pigs (*n* = 16). Also included are sow fecal (SF), sow vaginal (SV), sow colostrum (SC), and soy milk (SM) samples. *P < 0.05, **P < 0.01.

Similar to its effect on the piglet fecal microbiome, florfenicol had the greatest effect on the structure of the resistome on day 4 (Fig. S3; PERMANOVA: *R*^2^ = 0.25, *P* < 0.05). As may be expected, ARGs conferring resistance to the phenicols were relatively more abundant in the pigs treated with florfenicol from day 4 through 28 (Fig. 4; *P* < 0.05). However, there was also evidence that florfenicol was co-selecting for resistance to other antimicrobial classes as ARGs that confer resistance to aminoglycosides, beta-lactams, peptides, and sulfonamides were relatively more abundant in the metagenomes of treated piglets during at least one sampling period between 4 and 21 days of age. Conversely, ARGs conferring resistance to the macrolides-lincosamides-streptogramin B (MLS_B_) class were relatively more abundant in the control piglets on days 4 and 7, although the opposite was observed at day 84. The abundance of MLS_B_ and tetracycline ARGs in the pig feces remained relatively consistent from day 1 through 140, similar to the levels observed in the sows (Fig. 4).

**Fig 5:**
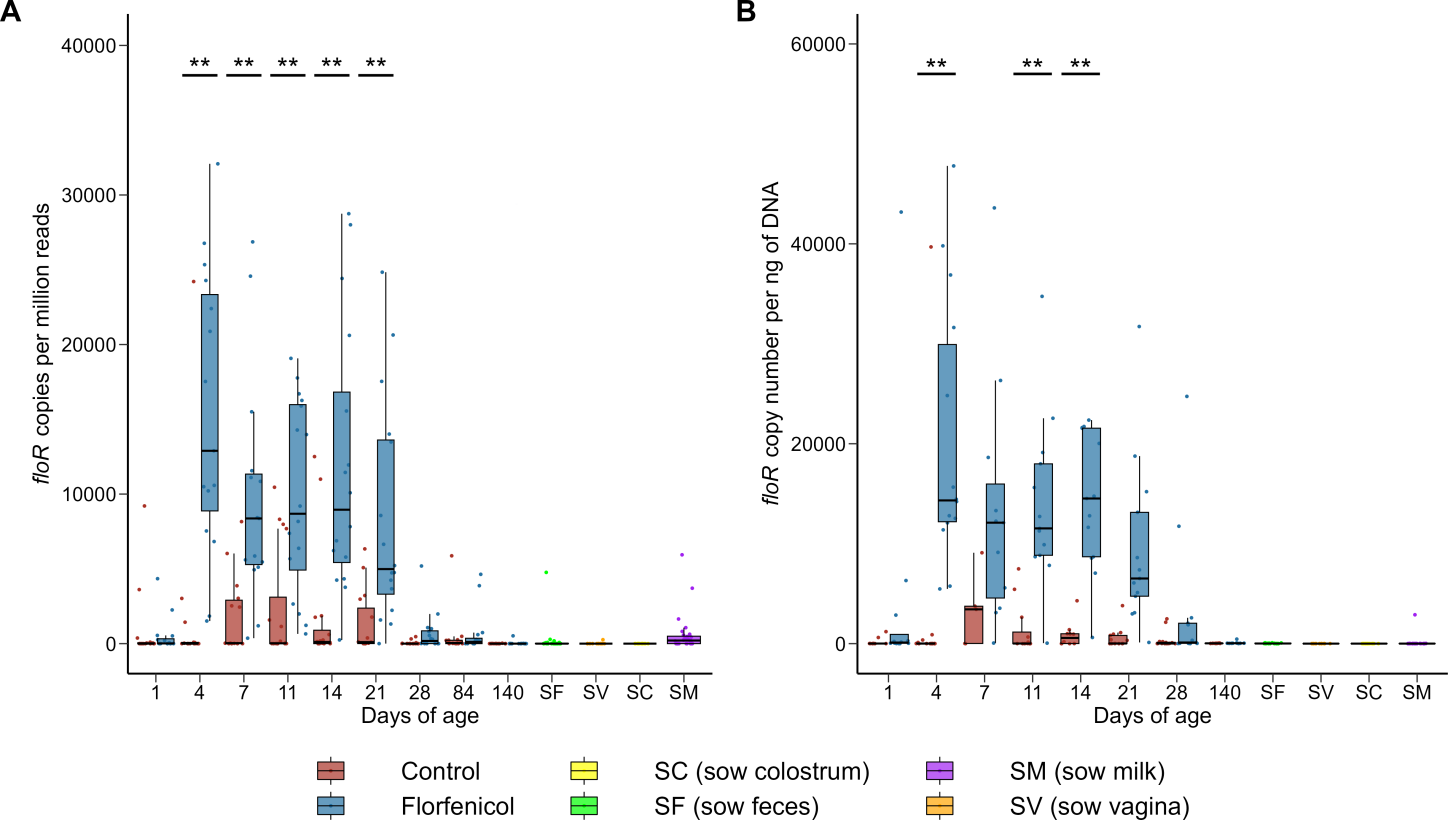
The relative abundance (**A**) and concentration (**B**) of the *floR* gene determined by metagenomic sequencing and qPCR, respectively, in the feces of control (*n* = 16) and florfenicol-treated (*n* = 16) pigs. Also included are sow fecal (SF), sow vaginal (SV), sow colostrum (SC), and soy milk (SM) samples. **P < 0.01.

In terms of individual ARGs, 111 were differentially abundant in the piglet fecal metagenomes on day 4, of which 91 were higher in relative abundance in the florfenicol-treated group (Table S5). The *floR* and *fexB* genes, which confer resistance to florfenicol, were most strongly associated with the florfenicol-treated piglets. These two ARGs were also relatively more abundant in the feces of the florfenicol-treated piglets from day 4 through 21. We also quantified the *floR* gene using qPCR, and the results largely mirrored that of the metagenomic data with the exceptions of days 7 and 21 (Fig. 5). The means of the two groups were clearly separated on these days. However, insufficient DNA was available for many of the samples from these two time points after 16S rRNA gene and metagenomic sequencing and therefore statistical power was reduced. Another florfenicol-specific resistance gene, *fexA*, was also more abundant in the piglets treated with florfenicol on day 4 to 14. The *fexA*, *fexB*, and *floR* genes all encode for phenicol-specific efflux pumps. The ARGs *cfr*, *cfr*(B), and *clc*D encode 23S ribosomal RNA methyltransferases that confer resistance to phenicols, along with other antimicrobial classes such as MLS_B,_ oxazolidinones, and pleuromutilins (63). Similarly, resistance to phenicols, as well as oxazolidinones, can be mediated through the ABC-F subfamily ATP-binding ribosomal protection protein genes *optrA* and *poxtA* (64). All five of these ARGs [*cfr*, *cfr*(B), *clcD*, *optrA*, *poxtA*] were relatively more abundant in the fecal microbiomes of florfenicol-treated piglets during at least one sampling time from day 4 through 21.

In addition to *fexB*, *floR*, and *clcD*, *bla*_CMY-59,_ a class C beta-lactamase gene, and *tet*(A), a tetracycline efflux pump gene, were also consistently relatively more abundant in the piglets administered florfenicol on day 4 through 21. With the exception of *tet*(A) and *tet*(D), the relative abundance of several *tet* genes, namely, *tet*(B), *tet*(O), *tet*(O/M/O), *tet*(Q), *tet*(T), *tet*(O/M/O), *tet*(W/32/O), *tet*(X1), and *tet*(Z), was higher in the control piglets on at least one sampling time from day 4 to 21 (Table S5). Similarly, certain MLS_B_ resistance genes, including *erm* (33), *erm* (47), *erm*(X), *lnu*(C), *lnu*(*P*), *lsa*(C), and *vat*(E), were also relatively more abundant in the control piglets.

### Sow colostrum, milk, vaginal, and fecal microbiomes and resistomes

The sow colostrum, milk, vaginal, and fecal microbiomes were also characterized to assess the potential contributions of these sources to the piglet fecal microbiome and resistome. Based on metagenomic sequencing, the relatively most abundant archaeal/bacterial species in the colostrum were *Lactobacillus amylovorus*, *Methanobrevibacter* sp900769095, *Streptococcus thoraltensis*, *Prevotella* sp945863825, *Rothia nasimurium*, *Lelliottia chinensis*, and *Cutibacterium acnes* (>2% relative abundance; Table S6). In milk samples collected on days 7 and 21, the bacterial species *Rothia nasimurium* was relatively most abundant. Similar to the colostrum, the potentially beneficial bacterial species *L. amylovorus* was among the relatively most abundant microbes in the milk samples (≥2.6%). However, unlike the colostrum, *S. suis*, a known pathogen in pigs, was relatively abundant in the milk samples at both sampling times (≥2.8%). The vaginal microbiome of the sows during farrowing was largely dominated by *B. rossii* (30.6% ± 8.0% SEM), *Streptococcus thoraltensis* (7.2% ± 3.1%), *Veillonella caviae* (3.8% ± 1.2%), as well as *S. suis* (4.2% ± 2.7%). *Bacteroides fragilis* was relatively most abundant in the feces of sows during the nursing period along with *Arcanobacterium* sp028724905, *E. coli* (particularly on day 0), *Mobiluncus porci*, and *Prevotella faecis* (Table S6).

**Fig 6:**
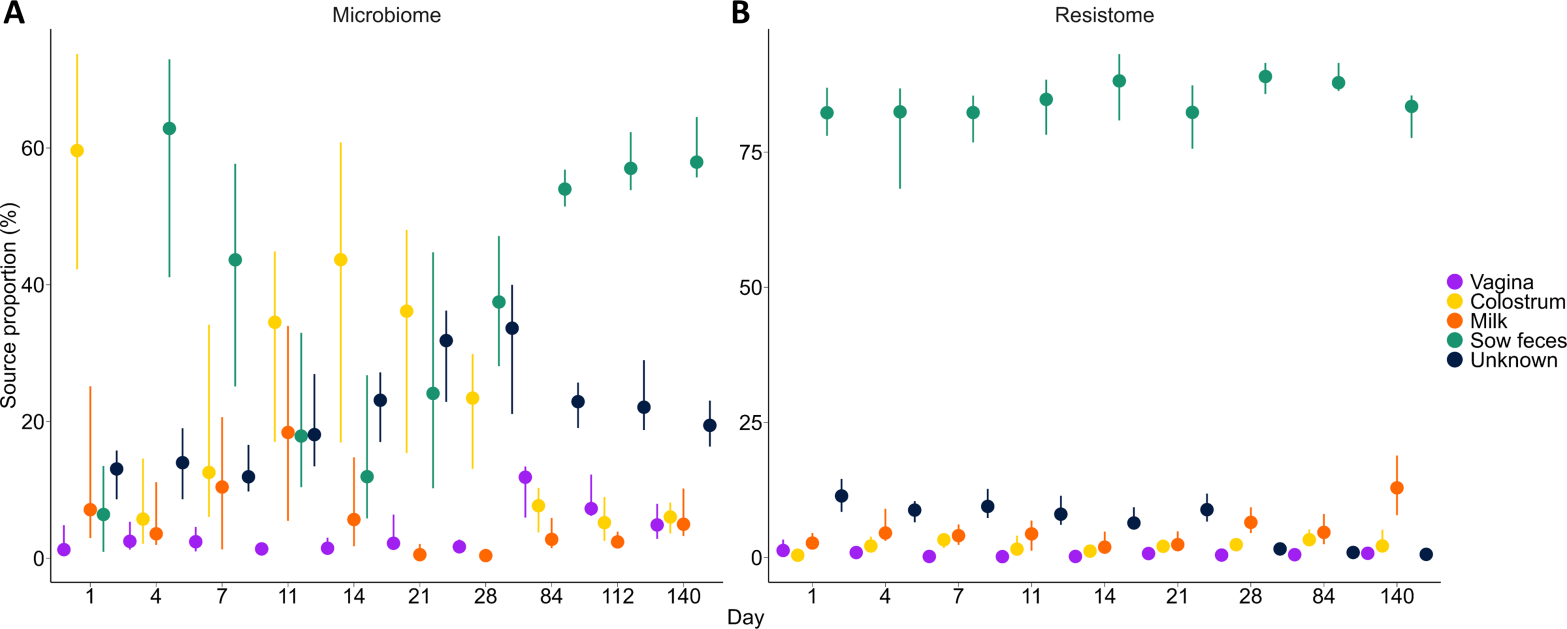
Predicted contributions of the vagina, colostrum, milk, and feces of the sows to the piglet gut microbiome (A; 16S rRNA gene sequences, *n* = 30) and resistome (B; metagenomes, *n* = 16). The points represent median values, and the lines are the 25th and 75th percentiles.

Using the 16S rRNA gene sequences of the control pigs and their sows, colostrum was identified as a major contributor to the piglet fecal microbiome, particularly on day 1 and up to day 28 (Fig. 6A). Sow feces were also predicted to be a significant source for the piglet fecal microbiome from day 4 through 140, although there was considerable variation especially in the nursing period. For the piglet resistome, sow feces were predicted to be the greatest source of ARGs throughout the entire production cycle (Fig. 6B). Although highly variable between samples, the colostrum, milk, and vagina carried ARGs conferring resistance to the MLS_B_, tetracycline, and beta-lactam classes (Fig. 4).

### Metagenome-assembled genomes

**Fig 7:**
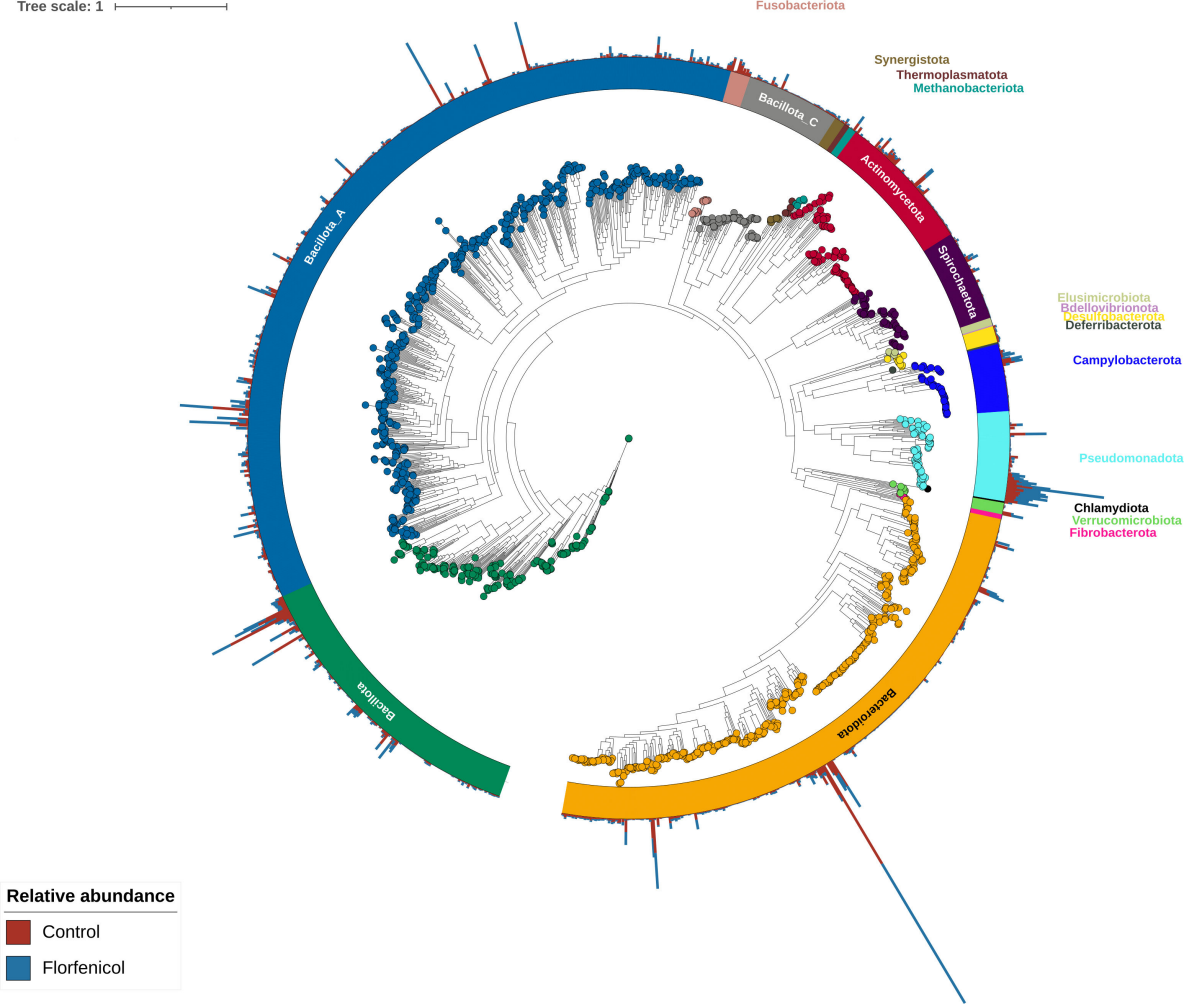
Maximum likelihood phylogenetic tree of the metagenome-assembled genomes (MAGs) recovered from pig fecal and sow colostrum, fecal, milk, and vaginal metagenomes based on the alignment of 400 marker genes. MAGs are colored and labeled by GTBD-tk assigned phyla. The outer bars display the overall percent relative abundance (0%–3.6%) of each MAG in the fecal microbiomes of control vs florfenicol-treated pigs.

A total of 1,546 non-redundant MAGs with less than 5% contamination and greater than 90% completeness were recovered from the piglet fecal and the sow colostrum, milk, fecal, and vaginal samples (Fig. 7; Table S7). The most frequently identified species represented by these MAGs were all uncultured species: *Sodaliphilus* sp004557565 (62 MAGs), *Collinsella* sp002391315 (29 MAGs), *Campylobacter* sp945873855 (24 MAGs), *Prevotella* sp000434975 (22 MAGs), and CAG-349 sp003539515 (Christensenellales, 19 MAGs). There were also 19 *E. coli* MAGs. Overall, *B. fragilis*, *Sarcina* (*Clostridium*) *perfringens*, *E. coli*, *Lactobacillus delbrueckii*, and *Limousia pullorum* MAGs were relatively most abundant in pig fecal samples, although there was considerable variation by sampling time (Table S7).

On day 4, a total of 209 MAGs were differentially abundant in the fecal microbiomes of the control (156 MAGs) and florfenicol-treated (53 MAGs) piglets (Table S8). Among the 53 MAGs that were relatively more abundant in the florfenicol-treated piglets were the 19 *E. coli* MAGs as well as MAGs classified as *Eisenbergiella porci*, *E. faecalis,* and *E. faecium*. All 29 *Collinsella* sp002391315 MAGs in the data set were relatively more abundant in the feces of the untreated piglets at this time as were 14 *Prevotella* sp000434975, 8 *P*. *hyovaginalis*, 7 *Desulfovibrio* sp900556755, and 7 *Peptostreptococcus porci* MAGs. Other notable MAGs that were relatively more abundant in the control piglet fecal microbiomes on day 4 were *Campylobacter coli*, *Clostridioides difficile*, *Streptococcus hyovaginalis*, and *S. suis*. There were 55 differentially abundant MAGs on day 7, 87 MAGs on day 11, 58 MAGs on day 14, 2 MAGs on day 21, and none on day 28. Only one MAG, higher in relative abundance in the control pig fecal samples and classified as *Peptoniphilus* sp022767005 (SUG3067), was consistently differentially abundant between the two groups of piglets from day 4 through 21. Interestingly, on day 84, there were 47 differentially abundant MAGs between the two groups of pigs, with 38 of these MAGs more relatively abundant in the control pigs. The majority of these MAGs (27) were classified as *Collinsella* sp002391315. At the next sampling time, day 140, only eight MAGs differed in relative abundance, and none were shared between days 84 and 140.

The MAGs were also screened for ARGs with 538 MAGs found to be carrying at least 1 ARG (Table S9). As expected, the 19 *E. coli* MAGs encoded the greatest number of ARGs as many of these genes are intrinsic to this species. The most widely encoded ARGs were *vanG* (206 MAGs), which typically confers only low-level resistance to vancomycin (65), and *adeF* (97 MAGs), a multidrug efflux pump gene. Although neither *fexB* nor *floR* were identified in any of the MAGs, *fexA* was binned into a *Staphylococcus borealis* MAG, and *ant(4′)-Ib*, *fexA*, and *optrA* were found in a MAG classified as *Vagococcus lutrae*. The *poxtA* gene was detected in 38 MAGs, 7 of which were relatively more abundant in the florfenicol-treated pigs during at least one sampling time. These seven MAGs included those assigned to *Eisenbergiella porci*, *Enterocloster clostridioformis*, *Enterocloster porci*, and *Hungatella hathewayi*. Genes conferring resistance to tetracyclines (*tet* genes) were identified in 152 different MAGs with *tet*(Q) (49 MAGs), *tet*(T) (36 MAGs), and *tet*(36) (35 MAGs) most prevalent. In addition, a *P. porci* MAG carried *cfr*(B) along with *lnu*(*P*) and a MAG designated *Clostridium* sp900759995 encoded *cfr*(B), *lnu*(C), and *tet*(36). *Clostridium* sp900759995 was relatively more abundant in the metagenomes of florfenicol-treated piglets on day 4, although the relative abundance of the *P. porci* MAG was greater in the untreated piglets on days 4 and 7.

### Florfenicol-resistant bacterial isolates

**Fig 8:**
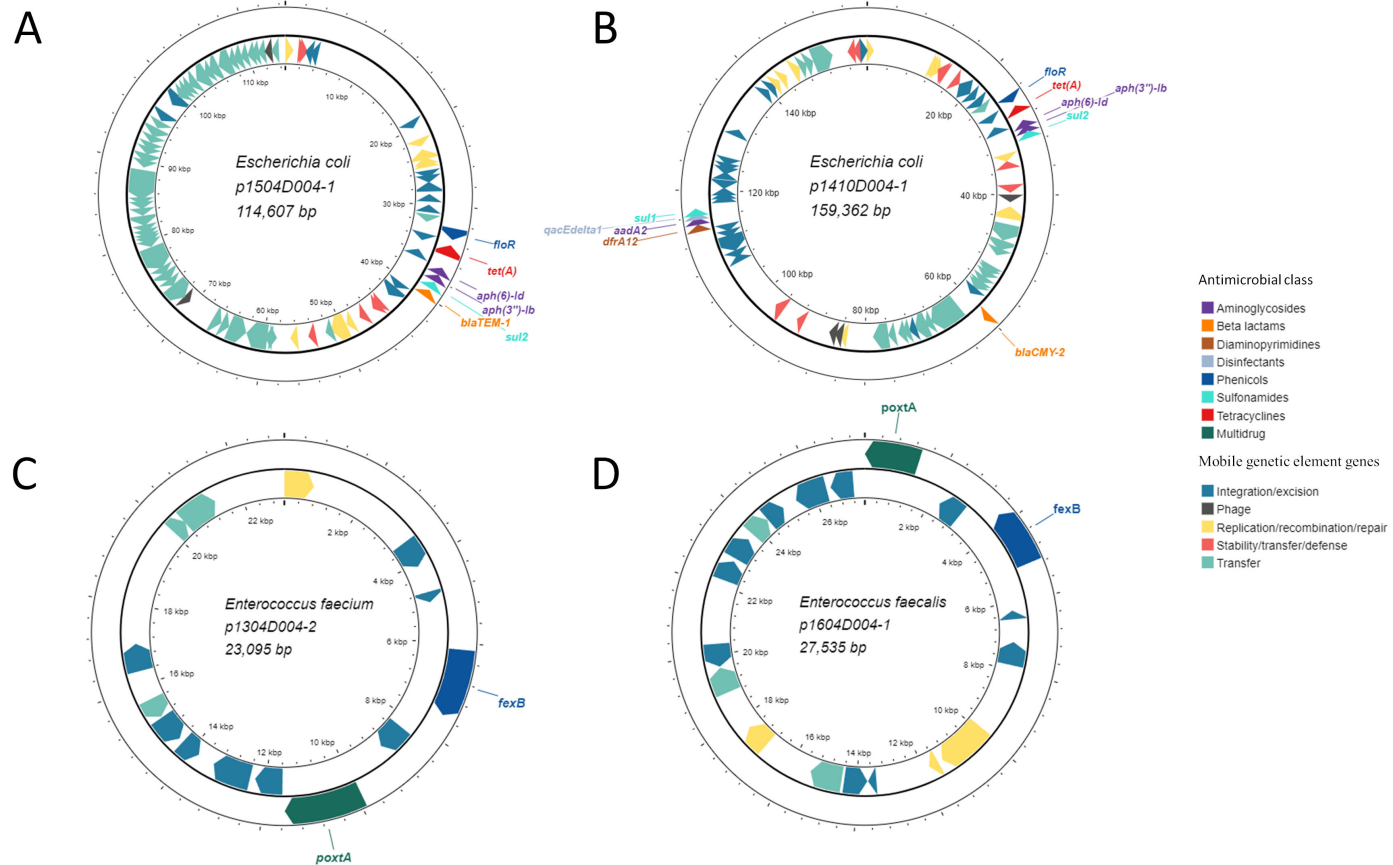
Locations of florfenicol resistance genes along with other antimicrobial resistance genes on plasmids in florfenicol-resistant *Escherichia coli* (**A and B**), *Enterococcus faecium* (**C**), and *Enterococcus faecalis* (**D**) isolates. The inner ring displays mobile genetic element genes colored by functional category.

To better understand the co-selection process, florfenicol-resistant bacteria (MIC > 32 µg/mL florfenicol) were isolated from fecal samples collected from florfenicol-treated piglets on days 4 and 11. These 11 isolate genomes were then sequenced with both short- and long-read sequencing technologies. The florfenicol-resistant isolates recovered were classified as *E. coli* (*n* = 7), *Enterococcus avium* (*n* = 1), *E. faecalis* (*n* = 2), and *E. faecium* (*n* = 1) (Table S10). The *E. coli* isolates were from three different serotypes: H49 (*n* = 3), O8:H25 (*n* = 3), and O4:H5 (*n* = 1). Using a 99.99% ANI threshold to define strains (66), we found that there were three different strains among the seven *E. coli* isolates, corresponding with the predicted serotypes (data not shown). Both *E. faecalis* isolates also appeared to belong to the same strain (>99.99% ANI, data not shown). Genes conferring resistance to florfenicol were found in all 11 isolates with the enterococci carrying *fexB* and the *E. coli* isolates encoding *floR*. The *floR* gene was located on a plasmid together with *aph(3″)-Ib*, *aph(6)-Id* (aminoglycosides), *bla*_TEM-1_ or *bla*_CMY-2_ (beta-lactams), *sul2* (sulfonamides), and *tet*(A) (tetracyclines) in the *E. coli* isolates (Fig. 8A and B). The *fexB* gene was co-located on a plasmid (23,095–27,535 bp) with *poxtA* in all four enterococci isolates (Fig. 8C and D).

This *fexB-poxtA* plasmid in both *E. avium* 1908D011 (p1908D011-2; 23,684 bp) and *E. faecium* 1304D004 (p1304D004-2; 23,095 bp) was 100% identical (100% coverage) to a plasmid (p1818-c; accession CP091209.1) in an *E. faecium* isolate from human feces in Switzerland (67) and was classified as a rep29 replicon (Fig. 8C; Table S10). *E. avium* 1908D011 also had a large plasmid (p1908D011-1; 129,571 bp) carrying *erm*(B) and *tet*(M) and with multiple replicons (repUS1 and repUS43) that was 99.9% identical (47% coverage) to a plasmid in an *E. faecalis* strain (accession LR961926.1). Another plasmid (p1304D004-4; 30,712 bp) in *E. faecium* 1304D004 encoded *tet*(L), *tet*(M), *ant (6)-Ia*, *lnu*(B), and *lsa*(E). In the two *E. faecalis* isolates, the *fexB-poxtA* genes were on a multireplicon (rep2 and rep6) Inc18 plasmid (Fig. 8D, p1604D004-1 and p1403D011-3) that was 100% identical (with 80% coverage) to a plasmid in an *E. faecium* strain recovered from cattle feces (68). To confirm that the *poxtA* gene was conferring the linezolid resistance phenotype, the MIC of linezolid for the four *Enterococcus* spp. isolates was determined. For both *E. faecalis* isolates, the MIC was 16 µg/mL, and for *E. avium* 1908D011 and *E. faecium*, the MIC of linezolid was 8 µg/mL. Based on CLSI breakpoints for linezolid and *Enterococcus*, these isolates were all resistant to linezolid. For the two ATCC strains without oxazolidinone resistance genes, *E. faecium* ATCC 35667 and *E. faecalis* ATCC 29212, the MIC of linezolid was 4 µg/mL.

In the six *E. coli* H49 and O8:H25 isolates, *floR*, *tet*(A), *aph(6)-Id*, *aph(3″)-Ib*, *sul2*, and *bla*_TEM-1_ were co-located on a ≈114,600-bp IncI1-I(Alpha) plasmid (Fig. 8A). In *E. coli* 1410D004, *floR*, *tet*(A), *aph(6)-Id*, *aph(3″)-Ib*, and *sul2* were in the same order on a plasmid (p1410D004-1) as in the other six *E. coli* isolates; however, *bla*_TEM-1_ was replaced by *bla*_CMY-2_, which was also farther downstream of these other ARGs (Fig. 8B). In addition, p1410D004-1 was larger (159,362 bp) and contained another cluster of ARGs downstream: *dfrA12* (diaminopyrimidines), *aadA2* (aminoglycosides), *qacEdelta1* (disinfectants), and *sul1*. None of the *E. coli* isolates met the 99.99% ANI strain threshold with any of the *E. coli* MAGs, although the H49 *E. coli* isolates had 99.95% ANI with SUG3627 (data not shown). Both *E. faecalis* genomes had 99.5% ANI with *E. faecalis* SUG2336 but only 98.4% ANI with *E. faecalis* SUG2337. The genome of *E. faecium* 1304D004 exhibited 99.8% ANI with *E. faecium* SUG2339, while the ANI between *E. avium* 1908D011 and the *E. avium* MAG SUG2338 was 98.3% (data not shown).

## DISCUSSION

Despite 20 years of use in Canadian swine herds and longer in other countries, relatively little is known about how florfenicol affects the swine fecal microbiome and resistome. Given the risk that antimicrobial resistance poses to both animal and human health, it is important to understand how antimicrobials such as florfenicol affect the pig fecal microbiome and resistome. Therefore, in this study, we assessed the impact of florfenicol treatment in piglets on the development of their fecal microbiome and resistome over the course of the swine production cycle. The fecal microbiome of piglets treated with florfenicol at 1 day of age and again at 7 days of age differed significantly from that of the control piglets from day 4 through 14. These differences were at least partly driven by a consistent enrichment of *Clostridium* spp., *Escherichia* spp., and CALYPF01 sp945271245 in florfenicol-treated piglets and *B. rossii*, *Fusobacterium* spp., *P. hyovaginalis*, and *H. porci* in the control piglets. Although florfenicol had the greatest effect on the fecal microbiome during the immediate periods following administration, there did appear to be some minor residual effects that persisted past the post-weaning phase, at least on day 84 of the study.

Considering that florfenicol is a broad-spectrum antimicrobial active against a number of different Gram-negative and Gram-positive bacteria, it may be expected that multiple bacterial species would be affected in the immediate period following treatment. Furthermore, as previously noted, florfenicol’s usage in pigs is comparatively recent and is primarily administered through intramuscular injection, unlike chlortetracycline, lincomycin, and tylosin, which are typically added to feed for consecutive days or weeks and which have been used in pigs for many decades (69, 70). As a result, the pig gut microbiome, which is vertically transmitted from sows to piglets across generations, has had comparatively less exposure to florfenicol and therefore less time to adapt or develop resistance. The relatively quick recovery of the piglet fecal microbiome to perturbation by florfenicol is likely due to the short half-life of florfenicol (5) and the fact that the piglet gut microbiome has yet to be stably established prior to weaning.

Not unexpectedly, florfenicol treatment increased the relative abundance of the florfenicol resistance genes *fexA*, *fexB*, and *floR*, as well as ARGs such as *cfr*, *cfr*(B), *clcD*, *optrA*, and *poxtA* that confer resistance to florfenicol and at least one other antimicrobial class (63). This increase in the abundance in the *cfr*, *clcD*, *cfr*(B), *optrA*, and *poxtA* genes in treated piglets is particularly noteworthy as these genes also facilitate resistance to the oxazolidinones. Oxazolidinones are classified by the World Health Organization as critically important in human medicine (9) and include linezolid, which is frequently used as an antibiotic of “last resort” against Gram-positive multidrug-resistant bacteria such as methicillin-resistant *Staphylococcus aureus* and vancomycin-resistant enterococci (71). Although *poxtA* has not previously been reported in Canada, this is probably due to the fact that it was only first described in 2018 (64). The greater abundance of *fexB* and *floR* in the fecal resistomes of florfenicol-treated piglets persisted until weaning, which was 14 days after the last treatment. Perhaps most significantly, florfenicol also increased the relative abundance of a large number of unrelated ARGs. Among these ARGs were those conferring resistance to aminoglycosides {*aadA8b*, *aph(3″)-Ib*, *aph(6)-Id*), beta-lactams (*bla*_CMY-59_), diaminopyrimidines (*dfrA12*), sulfonamides (*sul2*), and tetracyclines [*tet*(A)]}. Drugs within these antimicrobial classes are all used in swine in Canada via feed, injection, or water at varying frequencies (69, 70), and this likely explains why these ARGs initially emerged in the swine gut.

The MAGs recovered from the metagenomes were screened for ARGs to provide taxonomic context. In terms of florfenicol resistance genes, a *V. lutrae* MAG was identified carrying *fexA* together with *ant(4′)-Ib* and *optrA*, while a *S. borealis* MAG also carried *fexA*. However, neither the MAGs nor the bacterial species were differentially abundant between the two groups of pigs. Notably, multidrug-resistant *S. borealis* isolates with *fexA* and several other ARGs have recently been recovered from the nasal cavity of pigs (72). Similarly, *fexA* and *optrA* have been identified on the chromosome of *V. lutrae* isolated from the lung of a pig (73) and human feces (74). The *poxtA* gene, which is reported to provide resistance to tetracyclines in addition to phenicols and oxazolidinones (75), was detected in MAGs that were classified as *Anaerotignum* sp001304995, *Eisenbergiella porci*, *Enterocloster porci*, *E. clostridioformis*, or *H. hathewayi* and that were relatively more abundant in the metagenomes of florfenicol-treated piglets at day 4 or 7. This is in agreement with reports of *poxtA* in *H. hathewayi* (76) and *E. clostridioformis* (64) isolate genomes.

Overall, ARGs conferring resistance to tetracyclines and MLS_B_ remained largely unaffected by florfenicol treatment. However, there were certain ARGs within these classes that had a higher relative abundance in the control piglets at specific time points. Some of these ARGs such as *lnu*(C), *tet*(B), *te*t(T), *tet*(Q), and *tet*(W/32/O) were also binned into MAGs that were increased in relative abundance in the control piglets. For example, three *P. hyovaginalis* MAGs that were relatively more abundant in the metagenomes of the control pigs carried *tet*(W) and/or *tet*(W/32/O). Additionally, *tet*(Q) was binned into 27 *Collinsella* sp002391315 MAGs with a greater relative abundance in the control piglet microbiomes on day 4. This is consistent with reports of *tet* genes in certain *Collinsella* strains (77). Similarly, two *Basfia porcinus* MAGs carrying *tet*(B) were also relatively more abundant in the untreated pigs on days 4, 11, and 14. The type strain for this species (NCBI: *Actinobacillus porcinus* NM319), originally isolated from the pig respiratory tract, was reported to carry the *tet*(B) gene (78). It is therefore clear from this and earlier studies that ARGs conferring resistance to the MLS_B_ and tetracycline classes persist even in the absence of exposure to these antimicrobials (15, 24, 79, 80).

Although MAGs can provide genomic and taxonomic context for certain chromosomally encoded ARGs, many ARGs are not binned into MAGs, especially those on MGEs such as plasmids (81). Therefore, we isolated and sequenced the genomes of bacteria displaying phenotypic resistance to florfenicol (MIC > 32 µg/mL) that were recovered from the feces of treated piglets. The majority of these isolates were identified as *E. coli* (*n* = 7) from three different serotypes. All seven *E. coli* isolates carried *floR*, and most importantly, this gene was co-located on a plasmid with *aph(3″)-Ib*, *aph(6)-Id*, *bla*_TEM-1_/*bla*_CMY-2_, *sul2*, and *tet*(A). This likely explains the enrichment of these ARGs in the resistomes of piglets administered florfenicol, particularly since *E. coli* and other *Escherichia* spp. were also relatively more abundant in these piglets. Plasmids carrying these same ARGs have been identified in *E. coli* isolated from corvids (82), humans (83), pigs (84), dairy cattle manure (85), poultry (86), and groundwater (87). These ARGs have also been found on plasmids in other *Enterobacteriaceae* including *Salmonella enterica* subsp. *enterica* recovered from beef cattle (88) and their environment (89), as well as horses (88), humans (90), pigs (88, 91), and poultry (88), plus *Proteus mirabilis* from humans (92, 93). Thus, these ARGs appear to be widely disseminated together across multiple hosts and environments. Furthermore, it is probable that an antimicrobial from any of these classes may co-select for all of these ARGs.

The four enterococci isolates recovered from the feces of the florfenicol-treated piglets carried *poxtA* (and *fexB*) and were confirmed to be phenotypically resistant to linezolid. As with *floR* and other ARGs in the *E. coli* isolates, the *fexB* and *poxtA* genes were co-located on a plasmid in the *E. avium*, *E. faecalis*, and *E. faecium* isolates. The *fexB-poxtA* plasmid was nearly identical to ones found in *E. faecium* isolates from cattle (68), humans (67, 94), and pigs (95, 96) in Europe and China, indicating wide geographic and host distribution. Although nominally the *poxtA* gene confers resistance to tetracyclines, it is unclear whether this is true (75, 97). Additionally, oxazolidinones have never been approved for use in food-producing animals, and therefore, the presence of two co-located ARGs conferring resistance to phenicols is notable. The *fexB* and *poxtA* genes mediate phenicol resistance through distinct mechanisms, suggesting that the simultaneous presence of both genes results in higher levels of resistance to phenicols compared to the presence of only one gene.

Another objective of this study was to assess the potential transfer of bacteria and ARGs from the sows to their piglets. The piglet fecal microbiome is initially seeded with microbes from the vaginal tract during birth and from the colostrum they consume in the immediate postnatal period. Piglets are coprophagic, and as such, feces from the sow are also a significant source of bacteria for the piglet gut. In the present study, using source tracking, colostrum was predicted to be the largest contributor to the piglet fecal microbiome in the immediate post-farrowing period. This may be expected as piglets consume colostrum soon after birth, and this represents one of their first exposures to microorganisms. Many of the most abundant microbial species identified in the colostrum, such as *Methanobrevibacter* sp900769095, *Sodaliphilus* sp004557565, *Prevotella* sp900546535 (recently proposed name: *Segatella brasiliensis* [98]), and *Prevotella* sp000434975, are associated with the pig (99–101) or human GI tract (98). Therefore, although the teat was cleaned prior to sample collection, it is likely that the colostrum samples contained skin and fecal bacteria. Nonetheless, these are bacteria that the piglets would naturally be exposed to while suckling on the teat. The largest contributor to the piglet resistome was predicted to be the sow’s feces, with post-weaned pigs showing considerable overlap in their resistome with that of the sows. As mentioned, this was particularly evident for genes conferring resistance to the tetracycline and MLS_B_ classes.

Although the effect on the piglet fecal microbiome and resistome was largely lost post-weaning, it would be interesting to know if florfenicol would have these same effects if administered after weaning when the gut microbiome has primarily stabilized. In addition, although we demonstrate the potential for dissemination of certain florfenicol-selected ARGs, studies focused on evaluating horizontal gene transfer of these ARGs and the MGEs that harbor them would enhance our understanding of this process and help identify potential mitigation strategies. Also, this study would have missed identifying novel ARGs, as the focus here was on identifying known ARGs.

In summary, treatment with florfenicol in early life increased the abundance of ARGs conferring resistance not only to phenicols but also to several other antimicrobial classes as well. Furthermore, *E. coli* isolated from the feces of treated piglets contained a large plasmid with *floR* and other co-located ARGs providing resistance to at least four other antimicrobial classes. In addition, enterococci isolates carried *poxtA*, a multidrug resistance gene, on a plasmid together with *fexB*. Thus, this study shows the potential risk florfenicol use may pose in terms of co-selection and transfer of multiple ARGs.

## Data Availability

All metagenomic sequences, metagenome-assembled genomes, and isolate genomes are publicly available in the National Center for Biotechnology Information’s Sequence Read Archive and genome databases under BioProject PRJNA779404.

## SUPPLEMENTAL MATERIAL

Supplemental Figures msystems.01250-24-s0001.pdf Figures S1 to S3.

Supplemental Tables msystems.01250-24-s0002.xlsx Tables S1 to S10.

ASM does not own the copyrights to Supplemental Material that may be linked to, or accessed through, an article. The authors have granted ASM a non-exclusive, world-wide license to publish the Supplemental Material files. Please contact the corresponding author directly for reuse.
