# Supplementary material for: Florfenicol administration in piglets co-selects for multiple antimicrobial resistance genes
Source: mSystems. 2024 Nov 25;9(12):e01250-24. doi: 10.1128/msystems.01250-24 (PMC11651103; doi:10.1128/msystems.01250-24)
Supplement: Supplemental Figures — Figures S1 to S3. [file msystems.01250-24-s0001.pdf]

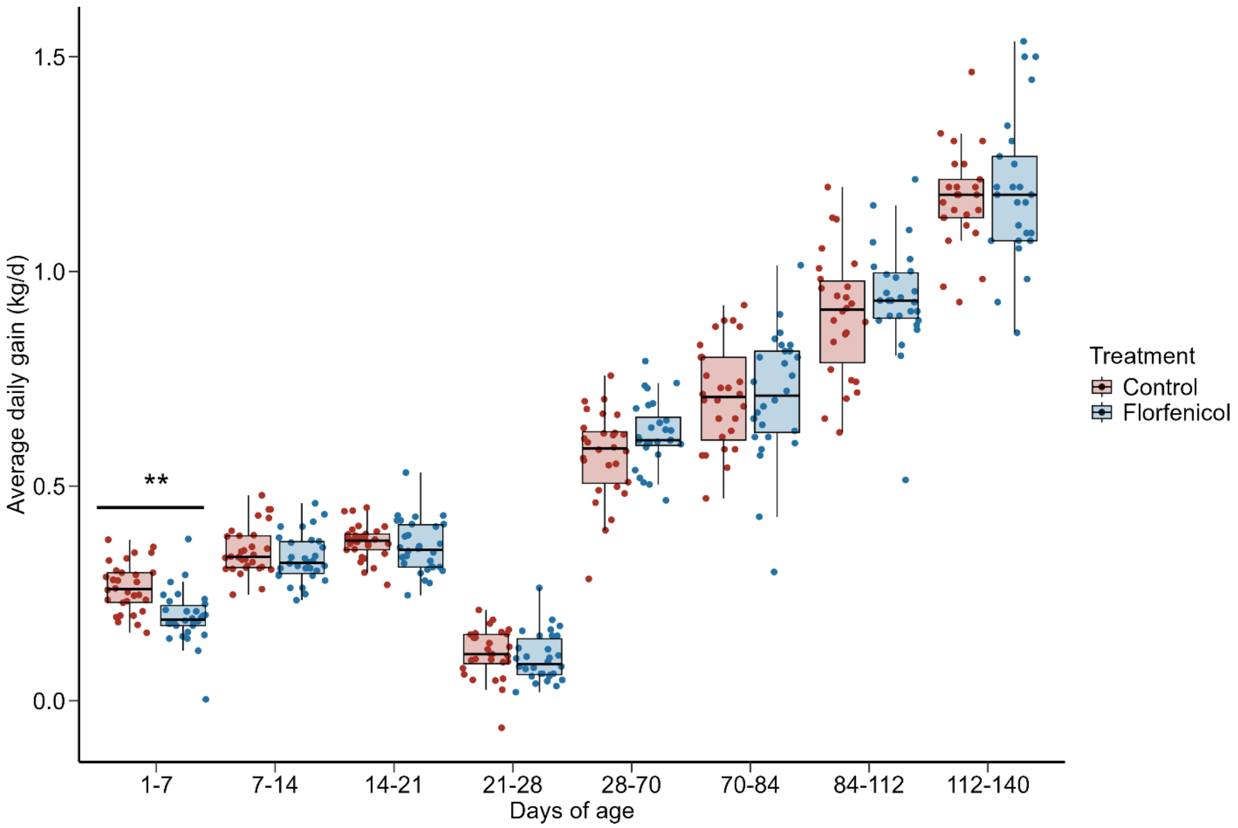

Supplementary Figure S1. Average daily gain for the control (n = 30) and florfenicol-treated (n = 30) pigs. ** = $P < 0.01$.

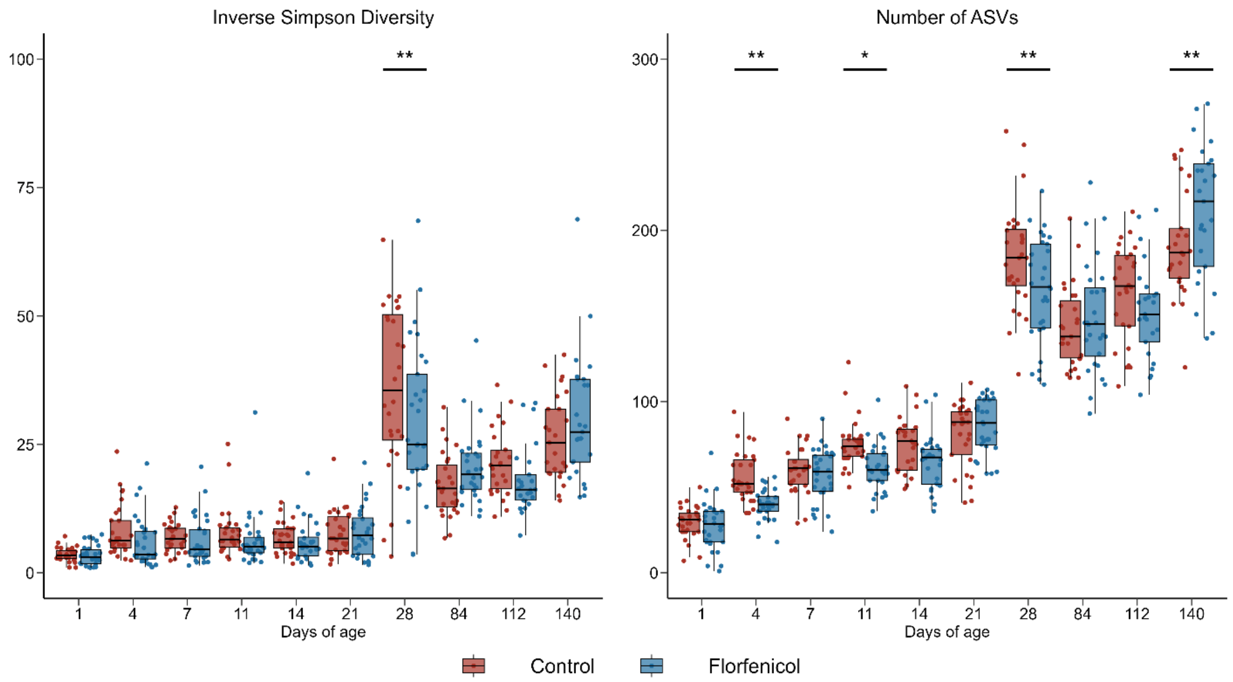

Supplementary Figure S2. Microbial diversity (inverse Simpson diversity index) and richness (number of ASVs) of control (n = 30) and florfenicol-treated (n = 30) pigs by sampling day based on 16S rRNA gene sequences. * P-value < 0.05; ** P-value <0.01.

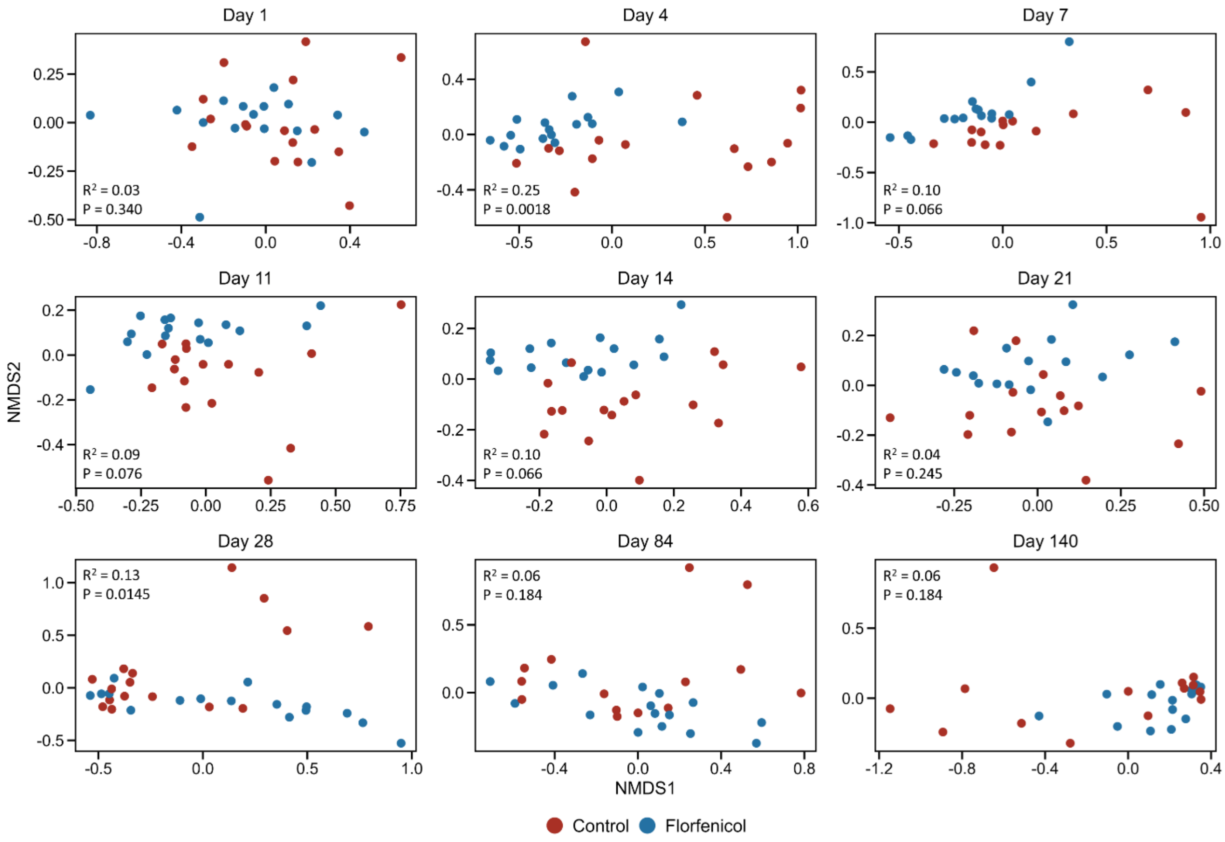

Supplementary Figure S3. Non-metric multidimensional scaling (NMDS) plots of the Bray-Curtis dissimilarities for untreated pigs (control; n = 16) and pigs treated with florfenicol (n = 16) on days 1 and 7 based on the relative abundance of antimicrobial resistance genes. Permutational multivariate analysis of variance (PERMANOVA) R^2 and P-values are included in each plot.
